# Supplementary material for: 28S rRNA-Derived Fragments Represent an Independent Molecular Predictor of Short-Term Relapse in Prostate Cancer
Source: Int J Mol Sci. 2023 Dec 23;25(1):239. doi: 10.3390/ijms25010239 (PMC10779029; doi:10.3390/ijms25010239)
Supplement: Supplementary file 1 [file ijms-25-00239-s001.zip › Supplementary Tables/Table S1.pdf]

Table S1. Descriptive statistics of 28S rRF levels in PCa and BPH patients.

Variables	Mean ± SE	Range	Percentiles				
			10	25	50 (median)	75	90
**PCa (n=89)**							
28S rRF	0.70 ± 0.44	<0.001-38.87	0.004	0.009	0.025	0.198	1.084
**BPH (n=53)**							
28S rRF	0.38 ± 0.07	<0.001-2.71	0.011	0.044	0.168	0.515	1.086

SE: Standard error.
