# Supplementary material for: 28S rRNA-Derived Fragments Represent an Independent Molecular Predictor of Short-Term Relapse in Prostate Cancer
Source: Int J Mol Sci. 2023 Dec 23;25(1):239. doi: 10.3390/ijms25010239 (PMC10779029; doi:10.3390/ijms25010239)
Supplement: Supplementary file 1 [file ijms-25-00239-s001.zip › Supplementary Tables/Table S2.pdf]

Table S2. Logistic regression analysis for the discrimination of PCa from BPH patients.

	Univariate Analysis			Multivariate Analysis[a]		
**Covariant**	**OR[b]**	**95% CI[c]**	***p*-value[d]**	**OR[b]**	**95% CI[c]**	***p*-value[d]**
**log (28S rRF)**	0.524	0.349-0.785	0.002	0.462	0.277-0.773	0.003
**PSA**	1.167	1.048-1.300	0.005	1.141	1.018-1.279	0.024
**Gender**	0.895	0.841-0.951	<0.001	0.917	0.856-0.982	0.014

[a] Multivariate logistic regression models adjusted for $\log_{10}$ 28S rRF, serum PSA and age.

[b] Odds ratio.

[c] Confidence interval of the estimated OR.

[d] Test for trend.
